# CCR2 overexpression promotes the efficient recruitment of retinal microglia in vitro

**Published:** 2012-12-14

**Authors:** Xiao-shuang Jiang, Ying-qin Ni, Tian-jin Liu, Meng Zhang, Hui Ren, Rui Jiang, Xin Huang, Ge-zhi Xu

**Affiliations:** 1 Department of Ophthalmology, Eye & ENT Hospital of Fudan University, Shanghai, People’s Republic of China; 2 Institute of Biochemistry, Chinese Academy of Science, Shanghai, People’s Republic of China

## Abstract

**Purpose:**

Retinal microglia can be activated and recruited by chemokines and play a protective role in early retinal degeneration. CC-chemokine ligand 2 (CCL2) and its receptor, CC-chemokine receptor 2 (CCR2), have been implicated as key mediators for the trafficking and accumulation of microglial cells in lesioned tissue. The current study investigates whether the overexpression of CCR2 allows microglia to migrate toward CCL2 more efficiently.

**Methods:**

Primary microglial cells were transduced with lentivirus carrying green fluorescent protein (GFP)-tagged CCR2 (CCR2-GFP). Overexpression of CCR2 was assessed by western blot analysis and fluorescence-assisted cell sorting. The chemotaxis of primary microglia transduced with lentivirus carrying CCR2-GFP was compared to either those transduced with GFP alone or those not transduced, using a chemotaxis chamber assay.

**Results:**

Primary microglia showed a high transduction rate following lentivirus application and maintained normal microglial morphology and a significant overexpression of CCR2 protein. We found that CCL2-mediated chemotaxis is concentration and time dependent in microglia. The chemotactic response of microglia cells overexpressing CCR2-GFP was significantly increased compared to that of nontransduced and GFP-expressing microglia.

**Conclusions:**

These findings suggest that microglia can be efficiently transduced with CCR2-GFP lentiviral vectors and that the overexpression of CCR2 in retinal microglia promotes their chemotaxis in response to chemokines, suggesting that these cells may be promising targets for cell-based therapeutic manipulation in retinal disease.

## Introduction

Microglia are considered to be specialized cells of the mononuclear phagocyte lineage and constitute the major resident immune cells in the central nervous system (CNS). Several reports indicate that microglia may be derived from two sources, the first being myeloid precursors that colonize the CNS, referred to as resident microglia [1-3]. In contrast, exogenous microglia precursors are derived from bone marrow cells (BMC) [4-11] or from circulating monocytes in the periphery [12,13]. Resident microglia have been shown to play dual roles in the progression of neurodegenerative disorders. These cells can release pro-inflammatory molecules that are neurotoxic and induce neurodegeneration [14,15]. However, microglia can also promote neuroprotection and neuroregeneration by entering CNS lesions and removing toxic byproducts and engulfing pathogens and cell debris to promote repair. In addition, microglia can release neurotrophic factors and anti-inflammatory molecules that induce the re-establishment of a functional neuronal environment [15,16]. However, the actions of the resident microglia alone are not sufficient to reverse neurodegenerative progression, and recent research has focused on the application of exogenous microglia precursors or monocytes to promote neuroprotection in the diseased brain and retina [10-13].

On the other hand, the number of exogenous microglia precursors that can be recruited from the periphery to the site of CNS lesions is too low to play a sufficient protective role. Large numbers (~10^7^ cells) of exogenous microglia precursors have been artificially administered to the periphery in mice, and only a small fraction of these transplanted cells are found to migrate to the CNS lesion site. Our objective was to test the hypothesis that enhancing the recruitment of transplanted microglia to the lesion site will significantly improve neuroprotection.

The recruitment of microglia recruited to lesion sites in the CNS, including the retina, is regulated by several molecules, among which chemokines and their cognate receptors are key players. The CC-chemokine ligand (CCL2) is the most potent microglia chemoattractant that binds CC-chemokine receptor 2 (CCR2) on microglia cell surfaces [17-23] and mediates the accumulation of these cells at sites of pathology [11-13]. This work represents the first to overexpress CCR2 in primary microglia via lentiviral transduction to enhance recruitment in response to CCL2. Our results indicate that retinal microglia can be efficiently transduced by lentiviral vectors, leading to high CCR2 expression. Importantly, our findings indicate that the augmentation of CCR2 expression in primary microglia enhances their chemotactic response to CCL2.

## Methods

### Cell culture

Newborn Sprague-Dawley rats were got from the SLRC Laboratory Animal Company (Shanghai, China) and treated in accordance with The Association for Research in Vision and Ophthalmology statement for the use of animals in Ophthalmic and Vision Research. Animals were humanely killed by intraperitoneal injection of ketamine (240 mg/kg) and xylazine (12 mg/kg). Eyes were dissected from postnatal day (P) 3 Sprague-Dawley rats, enucleated, hemisected, and the lens and vitreous were removed. The retinas were carefully isolated, avoiding contamination from the pigmented epithelium. Retinas were washed with 0.01M phosphate buffered saline (PBS; Hyclone, Logan, UT) then incubated in 0.25% trypsin EDTA (Gibco, Invitrogen, Carlsbad, CA) for 5 min at 37 °C. Following the inactivation of the trypsin by the addition of Dulbecco’s Modified Eagle Medium (Nutrient Mixture F-12 [DMEM/F12; Hyclone] medium containing 15% fetal bovine serum [FBS; Gibco] and 1% penicillin-streptomycin [Gibco]), the retinal pieces were dissociated by trituration and centrifuged. Retinal cells were plated in 75 cm^2^ flasks (Nunc, Roskilde, Denmark) and cultured in DMEM/F12 at 37 °C in a humidified atmosphere of 95% air/ 5% CO_2_ with media exchanges every 3 days. Following two weeks of incubation, microglial cells were loosely adherent and suspended in the media. The microglia-enriched cultures were shaken gently and the cells harvested from the medium. Microglia cells obtained by this method were 95% pure.

### Recombinant CC-chemokine receptor 2 lentiviral vector design and production

Lentivirus was produced from co-transfection with four plasmids (pPsv-REV, pMDlg-pRRE, pMD2G, and the transfer plasmid vector encoding GFP and CCR2, kindly provided by Dr. Trono of Geneva University). CCR2 was amplified using the following primers; CCR2 forward primer 5′-TGC TCT AGA GAA GAC AAT AAT ATG TTA CC-3′, CCR2 reverse primer 5′-ATA GCG GCC GCT TAC AAC CCA ACC GAG ACC T-3′. Recombinant lentiviral vectors was harvested 72 h following cotransfection of the pPsv-REV (10 μg), pMDlg-pRRE (15 μg), the transfer plasmid (20 μg), and pMD2G (7.5 μg) into 293T cells cultured in DMEM (10% FBS). Transfections were performed using Lipofectamine (Invitrogen, Carlsbad, CA) with the manufacturer’s recommendations.

### In vitro lentiviral vector transduction efficiency

Microglial cells were seeded in 96-well plates (Corning, Corning, NY) at a density of 2×10^3^/well, and 0.5 ml DMEM/F12 with 1% penicillin–streptomycin and 15% FBS was added to each well. Primary microglia were cultured for 24 h. Viral particles at a multiplicity of infection (MOI) of 2.5, 5, 10, 20, 50, and 100 were added to the wells. Following incubation at 37 °C in 5% CO_2_ for 24 h, the virus-containing medium was removed and replaced with 0.5 ml fresh culture medium per well. The transduction efficiency was determined daily with an inverted epifluorescent microscope (DMI3000B; Leica, Wetzlar Germany) and was quantified by measuring the GFP-expressing cells as a percentage of the total number of cells visible. As CCR2-GFP is a fusion protein, GFP-expressing microglia also express the CCR2 protein. Primary microglia transduced with 5 MOI of the CCR2-GFP lentivirus were used in the following studies as the morphology of these cells, which exhibited a typical rounded cell morphology with a large cytoplasm and a cell body with one or two extensions, was most similar to the nontransduced primary microglia.

### Western blot analysis

Five days following lentiviral vector transduction, CCR2-GFP-expressing microglia were washed with PBS, resuspended in 100 μl ice-cold cell lysis buffer (20 mM Tris), and lysed by ultrasound at 4 °C for 20 s. The lysates were centrifuged at 15,777 × *g* for 10 min at 4 °C and the protein concentrations were determined by spectrophotometry (NanoDrop ND-1000, Thermo Scientific, Wilmington, DE). Proteins were separated by sodium dodecyl sulfate-PAGE using 5% stacking and 12% separating gels and were subsequently transferred to polyvinylidene difluoride membranes (PVDF; Millipore, Billerica, MA). Membranes were blocked in Tris buffer (TBS, 50 mM Tris, PH 7.5) containing 5% skim milk and then incubated overnight at 4 °C with primary antibodies (rabbit anti-CCR2, 1:1000 dilution, Abcam, Cambridge, MA). Following washes in TBS containing Tween-20 (TBST), goat anti-rabbit immunoglobulin (IgG)-horseradish peroxidase (HRP) secondary antibody (1:2000 dilution, Cell Signaling Technology, Danvers, MA) was applied and incubated for 1 h at 37 °C. Equal amounts of protein loading were confirmed by re-probing the membranes with the mouse anti-β actin-HRP (1:10,000 dilution, Abcam). Immunoblots were visualized by chemiluminescence (Pierce Biotechnology, Rockford, IL) with exposure to autoradiograph film (X-OMAT AR; Eastman Kodak, Rochester, NY).

### Immunofluorescence

Primary microglia were subcultured on sterile glass coverslips for 12–16 h, washed in PBS, fixed in 4% formaldehyde for 10 min at 37 °C, permeabilized in 0.2% Triton-X 100 for 10 min at room temperature, washed in PBS, and then blocked in 1% BSA in PBS for 20 min at room temperature. The cells were then incubated with primary antibodies (rabbit polyclonal anti-CCR2 1:100, Abcam; mouse monoclonal anti-Iba11:100, Abcam; and mouse monoclonal ant-ED1 1:100, Abcam) overnight at 4 °C. Cells were then washed in PBS and incubated with secondary antibodies (Texas-Red-conjugated anti-rabbit IgG 1:1000, Invitrogen; Texa-Red-conjugated anti-mouse IgG 1:1000, Invitrogen; and Fluorescein isothicyanate-conjugated anti-mouse IgG 1:1000, Invitrogen) in PBS for 40 min at 37 °C. Images were captured using confocal laser microscope (TCS SP2; Leica) and analyzed using Leica software.

### Flow cytometry

CCR2-GFP-tagged microglia and primary microglia were collected, washed with PBS, and pelleted by centrifugation at 301 × *g* for 5 min. Single-cell suspensions were obtained by homogenization through 40-μm nylon cell strainers (BD Falcon, Franklin, NJ). The two groups of single cells were collected and analyzed for GFP fluorescence, using fluorescence-assisted cell sorting on a flow cytometer (Beckman, Brea, CA).

### Chemotaxis assay

Chemotaxis assays were performed using the BD Falcon chemotaxis chamber (BD Falcon), using a polyethylene terephthalate membrane with an 8-μm pore size. Microglial cells suspended in DMEM/F12 were added to the upper chamber and cell-free DMEM/F12 to the lower chamber and incubated overnight. Lentivirus expressing CCR2-GFP or GFP alone was added to the upper chamber to transduce the microglia. Following one media change, various dilutions of CCL-2 (1, 10, 20, 100 ng/ml, which induce increasing degrees of inflammation in the lesion area of the retina; data not shown) were added to the lower chamber on the fourth day and incubated for 6, 10, or 24 h. Nonmigratory cells were then removed from the membrane surface in the upper chamber, using a cotton swab, and cells that had migrated to the lower surface of the membrane were fixed with 4% formaldehyde for 10 m and labeled with 4,6-diamino-2-phenyl indole (DAPI; Sigma, St. Louis, MO) to visualize cell nuclei. The number of migrating cells was counted at 50X magnification, using a double-blinded approach, and images were acquired at 200X, using an epifluorescent microscope (LEICA DM 4000B; Leica)

### Statistical analysis

The number of transmigrated cells, those that migrated from the upper to the lower surface of the membrane, represents the chemotactic response of the cells. Data are presented as mean±standard error (SEM). Data from the different conditions were analyzed using one-way ANOVA for significant differences. Results were considered as significant at p<0.05.

## Results

### Primary culture of retinal microglia and immunocytochemical characterization

**Figure 1:**
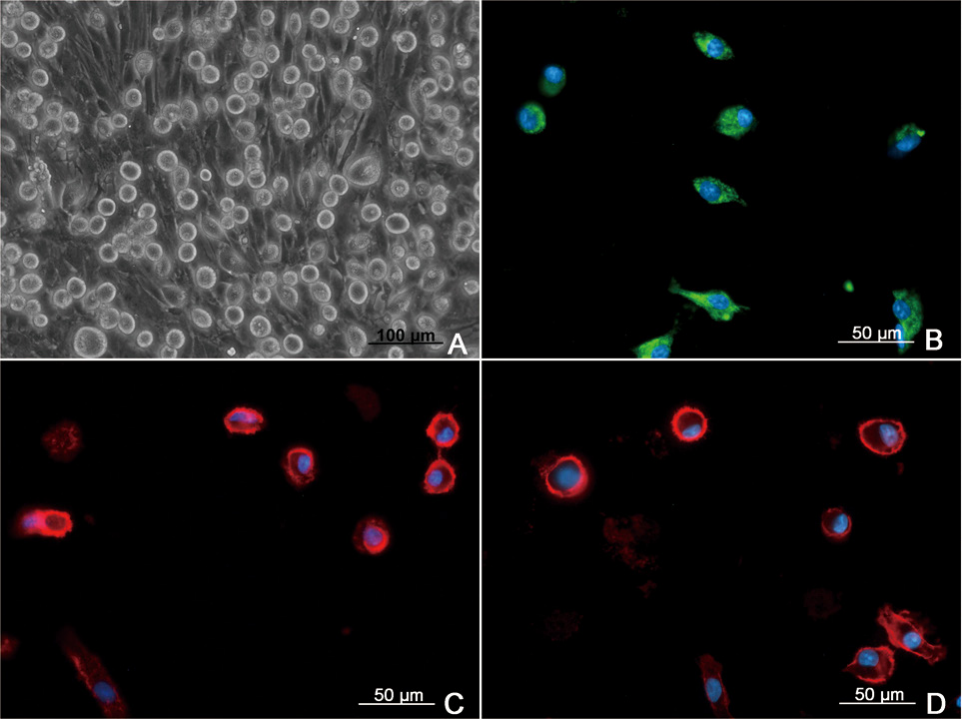
Identification of primary microglial cells. **A**: Primary microglial cells were harvested from 14-day-old microglia-enriched cultures from retina of P3 Sprague-Dawley rats (200X). **B-D**: Immunofluorescence showed the isolated cells can express microglial specific marker ED1, Iba1, and CD11b.

Retinal microglia were obtained from 14-day-old primary mixed glial cell cultures (Figure 1A) prepared from P3 Sprague-Dawley rats, using a “shaking off” method that the microglia were harvested by shaking the flasks at 301 × *g* for 1 h on an orbital shaker [24]. Immunofluorescence showed that the harvested cells were immunoreactive for the microglial-specific proteins ED1, Iba1, and CD11b (Figure 1B,C,D).

### Transduction efficiency

**Figure 2:**
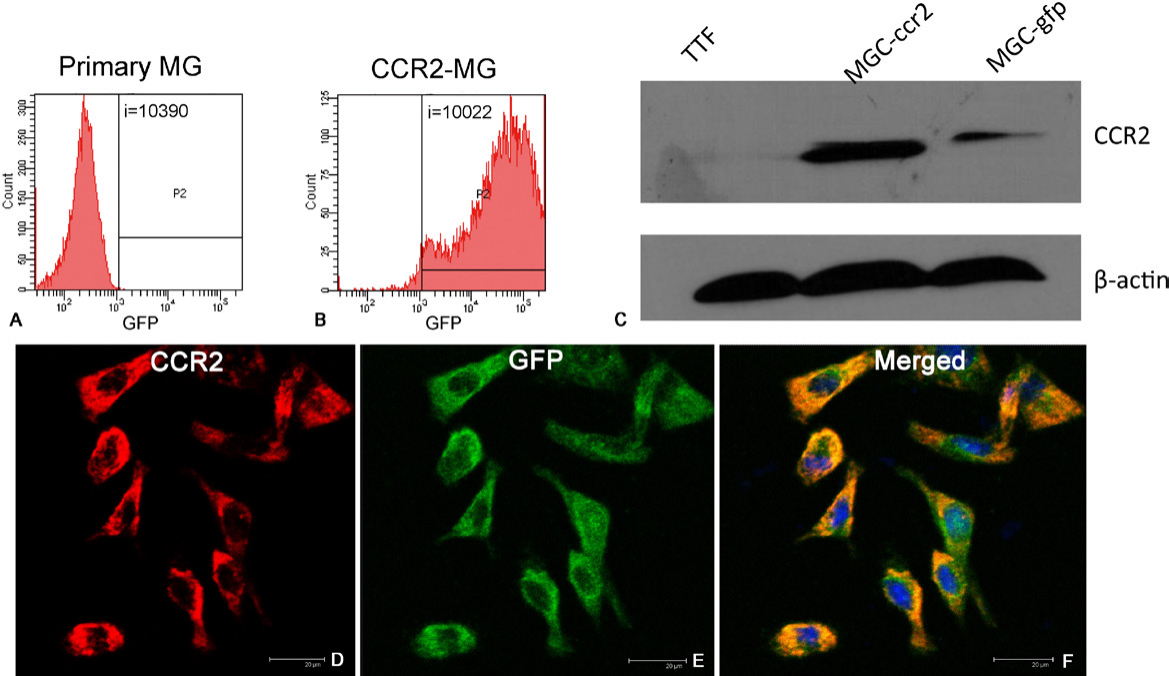
Identification of microglia transduced with green fluorescent protein (GFP)-tagged CCR2 (CCR2-GFP) lentivirus. **A**: Flow cytometric analysis showed untreated primary microglia were used as controls. **B**: Transduction efficiency was analyzed by flow cytometry for GFP fluorescence and reached 97.6%. **C**: Western blotting shows that microglia transduced with CCR2-GFP (MGC-ccr2) expressed more CCR2 than microglia transduced with GFP (MGC-gfp). TTF was tail tip fibroblast of mouse and was a negative control. **D-F**: Microglia transduced with CCR2-GFP express CCR2 and GFP.

**Figure 3:**
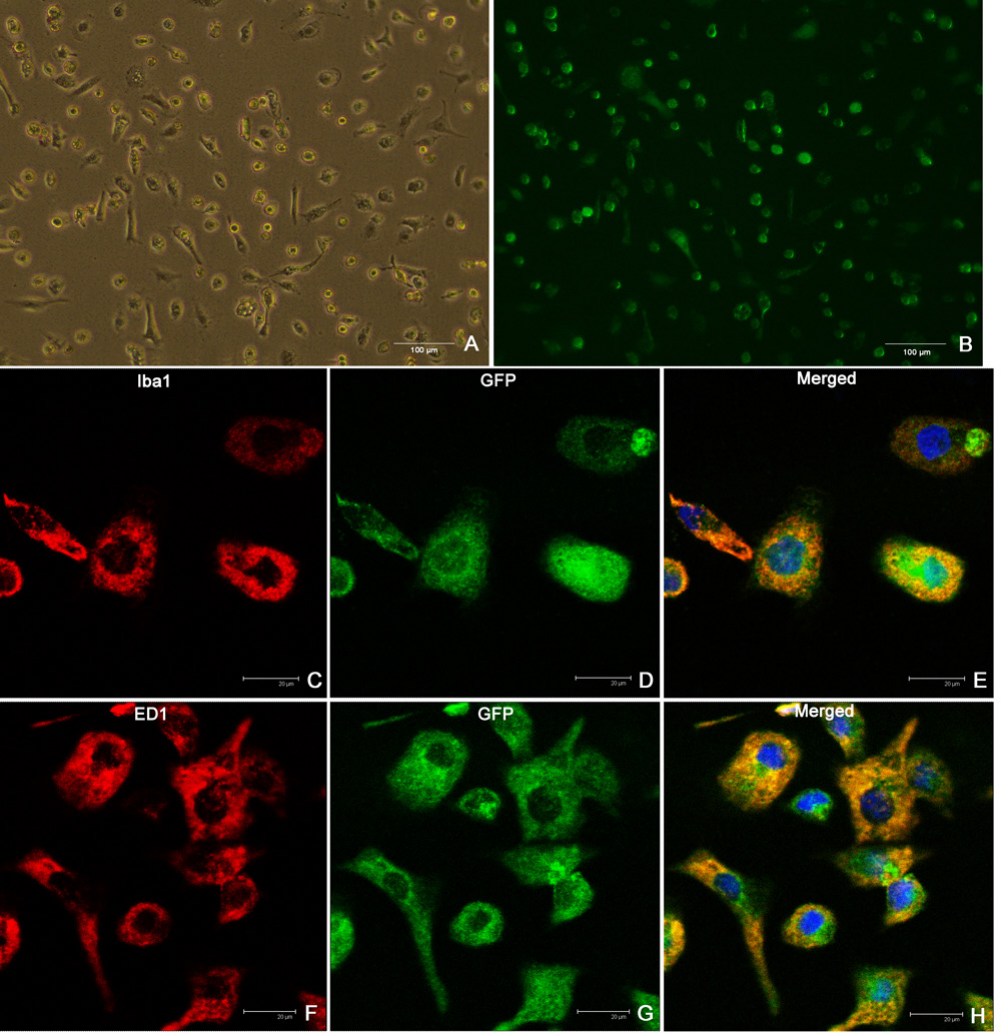
Microglia transduced with CC-chemokine receptor 2 (CCR2)-green fluorescent protein (GFP) lentivirus express microglia marker. Some cells are spindle shaped and a few are ramified; both shapes represent the “silent” state of microglia, while approximately 50% of primary microglia showed amoeboid or round shapes, indicating a state of activation. This culture composition is very similar to that of the untreated primary microglia. **A**: Microglia transduced with CCR2-GFP are shown under light microscopy. **B**: Microglia transduced with CCR2-GFP express green fluorescence. **C- H**: Microglia transduced with CCR2-GFP express microglia marker ED1 and Iba1 in vitro.

To investigate the transduction efficiency of the lentiviral vectors, primary cells were seeded in 96-well plates at 2×10^3^ cells per well and transduced at different MOIs. After 3 days nearly 99% of the transduced primary microglia were GFP positive at the MOIs of 50 and 100, whereas 55% of the cells were GFP positive at the MOIs of 5, 10, and 20. At MOI 2.5, 25% cells were GFP positive. On the fourth day following transduction, the GFP-positive microglia that had received lentivirus at MOI 100 and 50 began to aggregate and die by apoptosis. At the same time microglia transduced with MOI 5, 10, or 20 of lentivirus continued to expand. At MOI 5, the morphology of GFP-positive microglia was homogeneous, similar to that observed for nontransduced primary microglia. Consequently, a MOI of 5 was selected for the rest of the study. The green fluorescence of GFP and CCR2-GFP-expressing microglia was measured using flow cytometry to assess transduction efficiency. Figure 2A, B shows transduction efficiencies as high as 97.6% in the cultured microglia. These CCR2-GFP-expressing microglia also expressed markers of microglia, Iba1 and ED1 (Figure 3C-H).

### Overexpression of CCR2 in transduced primary microglia

Lentivirus-transduced primary microglia were subjected to western blot analysis to quantify CCR2 protein expression. We confirmed that CCR2 protein was indeed overexpressed in the CCR2-GFP-transduced primary microglia compared to microglia transduced with GFP alone. The level of CCR2 expression in the CCR2-GFP microglia was approximately 7.4 times higher than control (Figure 2C). Immunofluorescence confirmed that the CCR2-GFP microglia expressed CCR2 and that these microglia exhibit a typical rounded cell morphology with a large cytoplasm (Figure 2D-F).

### The chemotactic response of CCL2-stimulated microglia is concentration and time dependent

**Figure 4:**
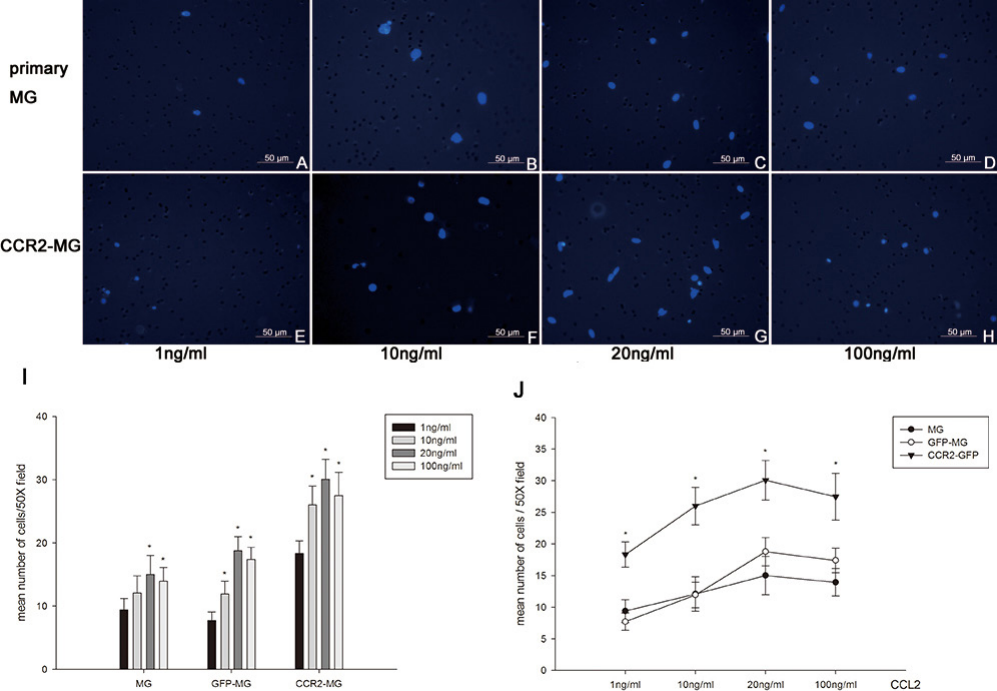
Concentration-dependent chemotactic response of CC-chemokine receptor 2 (CCR2) -transduced microglia and control in response to CC-chemokine ligand 2 (CCL2). Immunofluorescent images (**A-H**) showing the distribution of DAPI-labeled primary microglia (MG) and CCR2-GFP expressing microglia (CCR2-MG) that have passed through the membrane toward the chamber containing CCL2 at concentrations ranging from 1 to 100 ng/ml after 24 h in culture (200X). **A-D**: Primary microglia migrated to the lower surface of the chemotaxis chamber. **E**-**H**: CCR2-GFP-expressing microglia migrated to the lower surface of the chemotaxis chamber. **I**: Quantification of the chemotactic response of the various conditions of microglial cells (CCR2-MG, GFP-MG, and primary MG) to increasing concentrations of CCL2. The number of transmigrated cells for each condition represents the chemotactic capacity. Data represent the mean number of cells ±SEM from nine random fields. One-way ANOVA (*p<0.01) showed significant differences in chemotactic response at CCL2 concentrations of 10, 20, and 100 ng/ml of CCL2, while CCL2 at 1 ng/ml was indistinguishable from control (MG). **J**: Comparison of the chemotactic response of CCR2-MG, GFP-MG, and primary MG in the same conditions. The highest CCL2 concentration elicited submaximal effects, while the CCL2 at 20 ng/ml elicited maximal effects after 24 h treatment. Each experiment is representative of three independent experiments.

**Figure 5:**
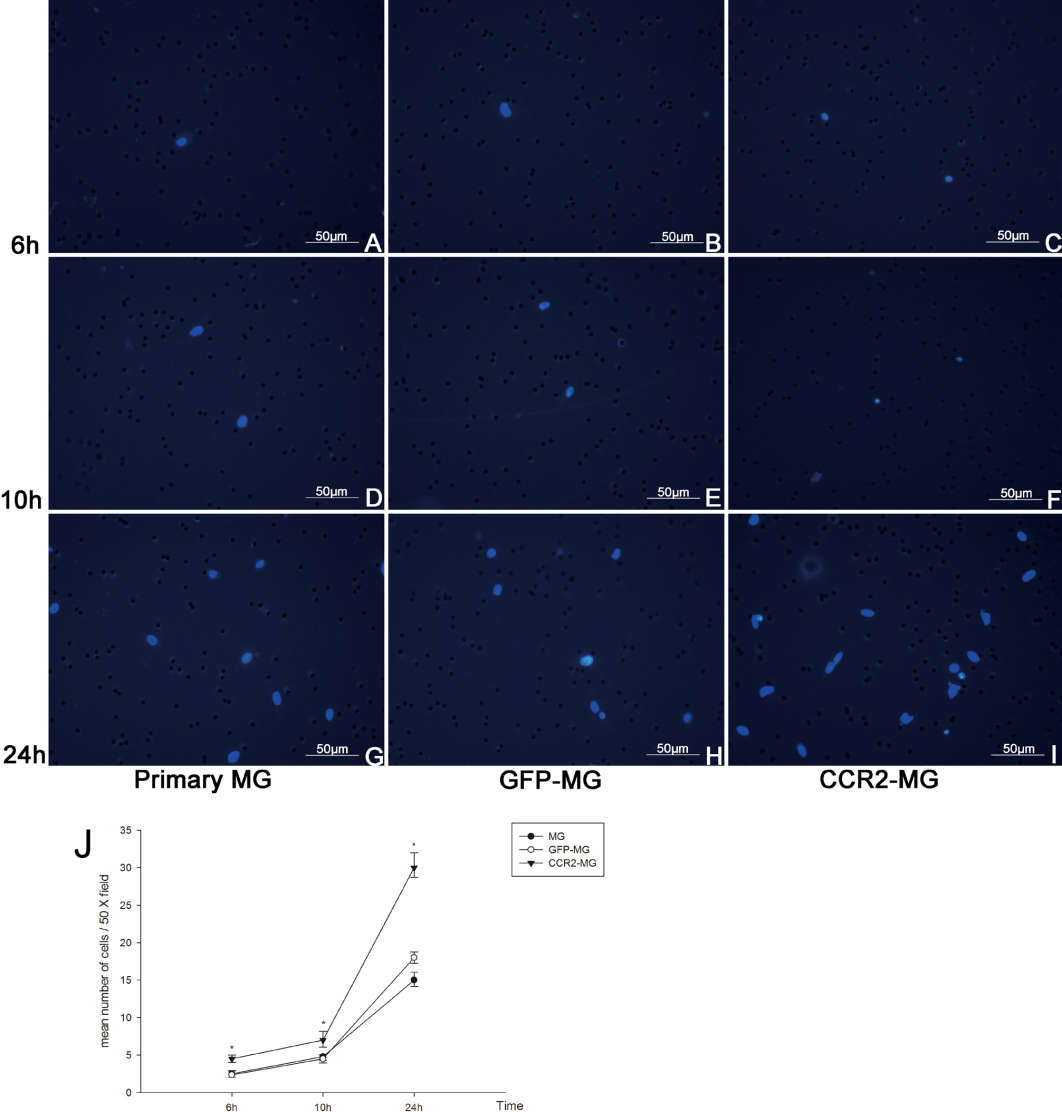
Time-course of microglial chemotaxis in response to CCL2. Immunofluorescent images (**A-I**) showing the distribution of DAPI-labeled microglia that have passed through the membrane after 6, 10, and 24 h of CCL2 exposure (200X). **A, D, G**: Primary microglia migrated to the lower surface of the chemotaxis chamber. **B**, **E**, **H**: GFP- expressing microglia migrated to the lower surface of the chemotaxis chamber. **C**, **F**, **I**: CCR2-GFP-expressing microglia migrated to the lower surface of the chemotaxis chamber. **J**. Quantification of the chemotactic response of various groups of microglia (CCR2- MG, GFP-MG and primary MG) at various time points (6, 10, or 24 h) following CCL2 exposure (20 ng/ml). Each value corresponds to the mean ± SEM from nine random fields. One-way ANOVA (*p<0.01) shows significant differences between CCR2-MG compared with GFP-MG and/or primary MG at the same time points. Each experiment is representative of three independent experiments.

Microglial cells were placed in the upper wells of specialized culture chambers, and their transmigration toward the bottom of the chamber, which contained CCL2 (1 ng/ml to 100 ng/ml), was assessed. Following incubation, the number of migrating microglia was quantified by counting the cells that had passed through the filter. This number represents the chemotactic response of the microglia. The number of transmigrated microglia per field was significantly increased following incubation with CCL2 at concentrations of 20 ng/ml and 100 ng/ml compared with CCL2 at 1 ng/ml after 24 h exposure (p<0.01; Figure 4), representing an increasing chemotactic response of the microglia with increasing concentration of CCL2. The highest CCL2 concentration elicited submaximal effects, while CCL2 at 20 ng/ml elicited maximal effects (Figure 4J). To evaluate the time course of chemotaxis, microglia were treated with CCL2 (20 ng/ml) for 6, 10, and 24 h. These data suggest that the chemotactic response of microglia is significantly increased at 10 and 24 h following CCL2 exposure compared to 6 h (p<0.01; Figure 5).

### CCR2 overexpression promotes the efficient recruitment of retinal microglia

**Figure 6:**
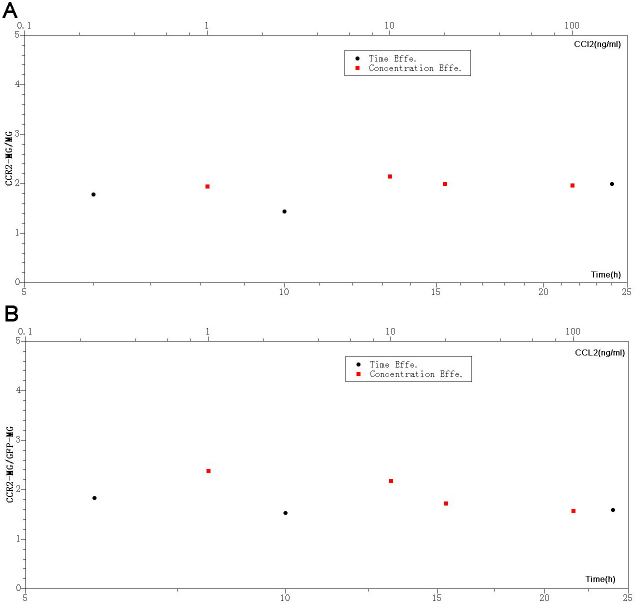
Chemotactic response in response to CC-chemokine ligand 2 (CCL2) increases twofold in CCR2-overexpressing microglia. The number of transmigrated cells in each condition represents the chemotactic response of the microglia. The number of migrating microglia overexpressing CCR2 (CCR2-MG) is approximately twofold compared to untreated microglia (MG; **A**) and GFP-transduced microglia (GFP-MG; **B**) at the same CCL2 concentration and posttreatment time points.

To determine whether overexpression of CCR2 could enhance the recruitment of retinal microglia, primary microglia transduced with the CCR2-GFP lentivirus (CCR2-MG) were subjected to the chemotaxis assay. Similar to the previous findings using primary microglia, the chemotactic response of the CCR2-GFP-transduced microglia was enhanced in a concentration- and time-dependent manner compared to GFP-transfected (GFP-MG) and untreated microglia (primary MG; Figure 4 and Figure 5). The chemotactic response of CCR2-GFP overexpressing microglia was consistently approximately twofold greater than that of either GFP-transduced or untreated microglia at the same concentration and time duration (Figure 6).

## Discussion

Chemokine molecules regulate the movement of microglia [25]. Of these, CCL2, also known as monocyte chemotactic protein (MCP-1), is a crucial member of the CC chemokine subfamily that controls the recruitment of monocytes to sites of inflammation by binding its receptor, CCR2. CCR2-knockout mice were shown to have significant defects in monocyte recruitment, suggesting that CCL2-mediated CCR2 activation is critically involved in the accumulation of monocytes at lesions [26]. Therefore, the CCL2/CCR2 pathway potentially represents an excellent target to enhance the recruitment of microglia precursors from the periphery to lesions in the CNS. Microglia represent the primary immune cells in the CNS where they continuously inspect their environment and react to changes that could threaten homeostasis [27,28]. As surveillance agents, microglia need to travel to the locations where they are needed. Our previous study suggested that microglia migrate to the photoreceptor layer in response to photoreceptor dysfunction or to debris in the subretinal space of the retina [29]. Several studies have focused on the effects of exogenous microglia precursor transplantation in various pathological models, including those for Alzheimer disease, Parkinson disease, and multiple sclerosis. However, potential means by which the recruitment of microglia to the CNS may be enhanced have not been reported. Therefore, the current study focuses on promoting the ability of microglial chemotaxis to migrate to sites of CNS damage by overexpressing CCR2.

Recent studies have used adenovirus, adeno-associated virus, as well as lentivirus as vectors for microglial transduction [30-34]. Lentiviral vectors have been reported to be efficient mediators of transgene expression in microglial cells that do not result in microglial activation [30,32,35].

The current study used a lentiviral-based system to overexpress GFP-tagged CCR2 to enhance microglial response to the CCR2 ligand, CCL2. Our results show a transduction efficiency as high as 97.6%, which is greater than previously reported [35]. This different transduction efficiency is most likely due to the different species and tissues used. Balcaitis et al. showed that enhanced GFP-tagged lentiviral transduced cells did not show any release of pro-inflammatory mediators (e.g., nitric oxide, tumor necrosis factor-alpha, interleukin-6) and also observed no difference in transduced versus control cells upon stimulation with lipopolysaccharide/interferon-γ [35]. Consistent with these previous findings, we observed that the morphology of CCR2-transduced microglia was indistinguishable from that of nontransduced primary microglia. Further studies will be needed to verify the usefulness of transduced microglia in in vivo injuries in the CNS. For example, our future studies will aim to study the phagocytic functions of CCR2-transduced microglia in the light-injured retina of Sprague-Dawley rats.

CCL2 regulates the migration of endogenous microglia and blood-derived macrophages to inflammatory sites in the CNS [36-38]. Via binding with CCR2, CCL2 has been reported to induce changes in actin polymerization and the subsequent reorganization of the actin cytoskeleton, the formation of focal adhesions, and pseudopod extension, which all contribute to the migration of activated microglia [39-41]. The present study demonstrates a concentration- and time-dependent microglial recruitment in response to CCL2. At higher concentrations of CCL2 in the lower chamber, more microglia migrated through the membrane. However, at the highest CCL2 concentration (100 ng/ml), microglial migration toward the lower chamber was not improved. We speculate the receptor in microglia is saturated by co-binding with CCL2; this is consistent with previous studies [40]. In addition, we observed a significant difference between CCR2-MG and GFP-MG and/or primary MG at 6 h, 10 h, and 24 h of migration.

In the current study, increases in CCR2 protein expression in microglia led to stronger migratory activity in response to CCL2, indicating that the CCL2–CCR2 interaction plays an important role in microglia recruitment. El Khoury et al. found that transgenic Tg2576 amyloid precursor protein transgenic mice (Tg2576), a model of Alzheimer disease-like pathology, is also deficient in CCR2 and these Tg2576 mice display reduced microglial accumulation around brain plaques [26]. Thus, a combination of CCR2 overexpression and microglial recruitment may represent a promising strategy for neuroprotection in the CNS.

In summary, we have shown the effective expression of GFP-tagged CCR2 in primary microglia with the use of lentiviral vectors and that the CCR2-overexpressing microglia show an enhanced CCL2-mediated recruitment. In addition to CCR2 expression, the presence of GFP in transduced microglia cells will allow their clear discrimination for further study in vivo. It remains to be confirmed whether the CCR2-GFP microglia have the same enhanced recruitment in vivo. Future studies will use these methods of CCR2 gene delivery to microglia in vivo for research in animal models of neurologic disease.
